# The Infarct-Limiting Effect of Remote Ischemic Conditioning in Rats Is Not Affected by Aspirin

**DOI:** 10.1007/s10557-023-07541-1

**Published:** 2023-12-20

**Authors:** M. V. Basalay, James M. Downey, S. M. Davidson, D. M. Yellon

**Affiliations:** 1 The Hatter Cardiovascular Institute, University College London, 67 Chenies Mews, London, WC1E 6HX UK; 2 Department of Physiology and Cell Biology, College of Medicine, University of South Alabama, Mobile, AL 36688 USA

**Keywords:** Remote conditioning, Aspirin, Ischemia/reperfusion injury

## Abstract

**Purpose:**

Remote ischemic conditioning (RIC) has been shown to be a powerful cardioprotective therapy in animal models. However, a protective effect in patients presenting with acute myocardial infarction has failed to be confirmed. A recent pre-clinical study reported that aspirin which is routinely given to patients undergoing reperfusion therapy blocked the infarct-limiting effect of ischemic postconditioning. The present study was designed to test whether aspirin could also be blocking the infarct-limiting effect of RIC.

**Methods:**

This was investigated *in vivo* using male Sprague Dawley rats (*n* = 5 to 6 per group) subjected to either 30 min of regional myocardial ischemia, followed by 120-min reperfusion, or additionally to a RIC protocol initiated after 20-min myocardial ischemia. The RIC protocol included four cycles of 5-min hind limb ischemia interspersed with 5-min reperfusion. Intravenous aspirin (30 mg/kg) or vehicle (saline) was administered after 15-min myocardial ischemia.

**Results:**

RIC significantly reduced infarct size (IS) normalized to the area at risk, by 47%. Aspirin administration did not affect IS nor did it attenuate the infarct-limiting effect of RIC.

**Conclusion:**

Aspirin administration in the setting of myocardial infarction is not likely to interfere with the cardioprotective effect of RIC.

**Supplementary Information:**

The online version contains supplementary material available at 10.1007/s10557-023-07541-1.

## Introduction

Despite the rapid progress in the development of recanalization techniques and rapid improvement in accessibility of these techniques for patients with acute coronary syndromes (ACS) worldwide, the detrimental effects of reperfusion injury remain a major problem. Currently, remote ischemic conditioning (RIC) is a potential strategy for the alleviation of reperfusion injury which may be applicable to the clinical setting of ACS. The infarct-limiting effect of RIC has been demonstrated by many research groups and in different animal species [1]. The strength of this effect in pre-clinical research is comparable to that of classical ischemic preconditioning. It is understandable, therefore, that the potential of using RIC in clinical practice has been appealing. While a number of proof-of-concept clinical trials in patients with acute myocardial infarction have demonstrated heterogeneous but generally promising results on RIC’s ability to reduce myocardial damage [2], such protection was not confirmed by a recent large-scale, international, multicenter, randomized clinical trial [3]. This failure has raised an intense discussion on possible factors which may impede the cardioprotective effectiveness of RIC in patients [4]. One possible explanation is that the medications included in standard therapeutic protocols could be blocking RIC’s protection [4]. It has recently been reported that aspirin, a mainstay in the primary care in patients with ACS, blocks the cardioprotection from ischemic postconditioning in rats [5]. Platelets are known to be key players in cardioprotection [6]. They have recently been shown to transport a cardioprotective signal from a remote organ to the heart, and aspirin abolished the transfer of this protective signal with platelets to isolated rodent hearts [7].

The aim of the present study was to evaluate whether aspirin would attenuate the infarct-limiting effect of RIC in our animal model. If that proved to be the case, then it could explain why RIC is not as protective in patients as it is in animal models where aspirin is absent.

## Materials and Methods

All the experiments were performed in accordance with the European Commission Directive 2010/63/EU (European Convention for the Protection of Vertebrate Animals used for Experimental and Other Scientific Purposes) and the UK Home Office (Scientific Procedures) Act (1986) with project approval from the University College London Institutional Animal Care and Use Committee. The animals were group-housed and maintained on a 12-h light cycle (lights on 07:00) and had ad libitum access to water and food.

### Animals

Male Sprague Dawley rats of 190–210 g weight, *N* = 23 in total, were purchased from Charles River Laboratories and allowed to acclimatize under standard conditions for at least 1 week. The weight of the rats by the time of their inclusion into the experiment was 250–300 g.

### Ischemia/Reperfusion Model

**Fig. 1:**
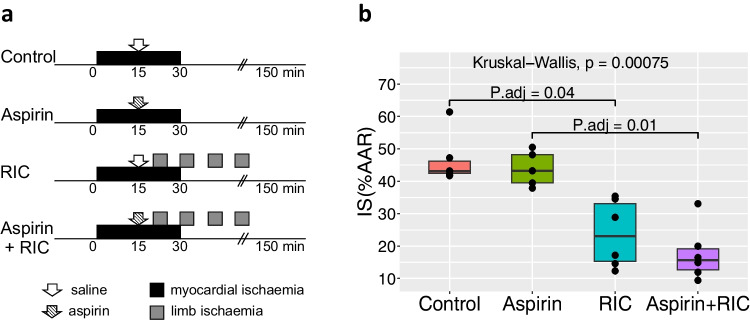
Aspirin does not attenuate the infarct-limiting effect of remote ischemic conditioning. **a** The experimental protocol. 30-min myocardial regional ischemia was followed by 2 h of reperfusion. Remote ischemic conditioning (RIC) was initiated on the 20th min of myocardial ischemia. It was performed as four 5-min cycles of limb ischemia, interspersed with 5-min intervals of reperfusion. Aspirin was dissolved in saline and administered into the left jugular vein as a 3-min bolus, finishing on the 15th min of myocardial ischemia. Control and RIC groups received the same volume of saline. The number of animals in the groups was the following: control (*n* = 6), aspirin (*n* = 5), RIC (*n* = 6), aspirin + RIC (*n* = 6). **b** Infarct sizes normalized to the areas at risk (IS(%AAR)) in the experimental groups. The scatterplot represents individual data for each animal, and the boxplot illustrates medians, 25th and 75th percentiles for the groups. The groups were compared using Kruskal-Wallis, followed by Dunn’s Multiple Comparison post hoc test. *P* values were adjusted using Bonferroni correction method (P.adj). Values of P.adj < 0.05 were considered statistically significant

Rats were anesthetized with isoflurane: 4% isoflurane for induction and 3–3.75% for maintenance. The maintenance dose was selected on the basis of complete absence of pedal reflex and spontaneous breathing, starting from 2.5% and being increased by 0.25% every 5 min if required. The rats were intubated, their right common carotid artery and left jugular vein cannulated, and the chest was opened by dissecting two ribs to the left of the sternum. The heart was then exposed using a chest retractor. After a 10-min stabilization period, the arterial blood was collected into a capillary tube to measure blood pH. If required, the parameters of ventilation were adjusted, with a re-check of the pH after 10 min. The left anterior descending coronary artery (LAD) was then ligated with a polypropylene suture, needle size 5–0, for 30 min after which it was reperfused for 2 h (Fig. 1a). Cling film was used to cover the opening of the chest throughout the experiment to prevent cooling and drying of the heart. Blood pressure and heart rate were recorded throughout the experiment, and body temperature maintained at 36.4 to 37.5 °C. At the end of the reperfusion period, the LAD was re-occluded and the hearts perfused, via the jugular vein, with 5% Evan’s blue dye to delineate the area at risk (AAR). The heart was then rapidly excised, the right ventricle removed, and the left ventricle frozen at −80 °C and sliced into 6–7 slices of equal thickness. Each slice was scanned from both sides to obtain the images of the AAR. After that, the infarcted myocardium was detected by incubating the frozen heart slices with 1% 2,3,5-triphenyltetrazolium chloride in Tris buffer (pH 7.4) for 15 min at 37 °C and fixing in 4% formalin for 24 h. Finally, the slices were scanned, again from both sides, and each slice was weighed. The AAR and infarct size (IS) were planimetrically evaluated using ImageJ (https://imagej.nih.gov/ij/). The weight of the AAR was normalized to the weight of the left ventricle. IS was expressed as a proportion of the weight of the infarcted myocardium to the weight of the AAR. The template used for calculations of the IS and the AAR is presented in the Supplement.

### Remote Ischemic Conditioning

RIC was initiated at the 20th min of ischemia and performed using four cycles of 5-min left hind limb ischemia interspersed with 5-min reperfusion, using an inflatable 12-mm cuff (IVM, USA), which was inflated to 200 mmHg and subsequently deflated (Fig. 1a). The efficiency of blood cessation with hind limb cuff inflation in rats had previously been confirmed.

### Drug Administration

The dose of aspirin of 30 mg/kg was chosen based on the previous studies, showing that 20 mg/kg of aspirin abolished the infarct-limiting effect of local ischemic postconditioning [5] and that this effect of aspirin was dose-dependent [8]. In our study, aspirin (Sigma-Aldrich) was dissolved in 1.5 ml of saline at 37 °C directly before each of the experiments. This solution was administered as an intravenous bolus over 3 min, finishing at the 15th min of ischemia (Fig. 1a). The groups not requiring the administration of aspirin received the same volume of saline warmed to 37 °C, over the same time window.

### Randomization and Blinding

Rats were randomly allocated to the experimental groups. The experimenter was not blinded to the experimental groups; however, IS was subsequently evaluated in heart slices by an investigator who was blinded to the treatment.

### Statistical Analysis

The obtained data were analyzed with RStudio (https://cran.r-project.org, version 2023.06.1). The normality of distribution within the groups was evaluated with Shapiro-Wilk, Kolmogorov-Smirnov, and Pearson tests. The groups were compared using Kruskal-Wallis test, followed by Dunn’s Multiple Comparison post hoc test with Bonferroni correction method. The non-parametric test was chosen based on the presence of outlying IS values in two groups (Fig. 1b), as well as due to the small number of animals (5 to 6) per group. Adjusted values of *P* < 0.05 (P.adj) were considered statistically significant. Data are reported as median, 25th and 75th percentiles (IQR). The format of presenting the data is: median [IQR, 25th–75th percentile].

## Results

None of the animals died prior to completion of the protocol nor were any excluded. There were no differences in hemodynamic parameters between the groups at any time point of the experimental protocol. AAR was also comparable in all the experimental groups. All the raw data are presented in Supplement.

IS in the control group was 43% [IQR, 42–46%] (Fig. 1b). RIC, as expected, reduced IS: 23% [IQR, 15–33%] (P.adj < 0.05 vs. control). Aspirin alone had no effect on IS: 43% [IQR, 39–48%]. Similarly, aspirin had no effect on the infarct-limiting effect of RIC (aspirin + RIC group): IS = 15% [IQR, 12–19%] (P.adj < 0.05 vs. aspirin).

## Discussion

Platelets are known to play the key role in the pathophysiology of ACS, as their activation and aggregation contribute to initiation and propagation of acute ischemia/reperfusion injury. However, healthy platelets may also activate the cardioprotective RISK pathway (reviewed in [9]), a universal signaling pathway shared by a number of cardioprotective therapies, including ischemic postconditioning [10]. Interestingly, aspirin has recently been demonstrated to abolish the infarct-limiting effect of local ischemic postconditioning in a rat model of ischemia/reperfusion [5]. The exact mechanism of this has not been investigated. We believe that RISK pathway inhibition by aspirin may be one of the plausible explanations. Interestingly, in another study, the infarct-limiting effect of classical ischemic preconditioning was not affected by aspirin [11]. However, the dose of aspirin in that study was half that used by Birnbaum et al. This may be important, as the attenuation of statin-induced cardioprotection by aspirin was shown to be dose-dependent [8]. In addition, the infarct-limiting effect of classical ischemic preconditioning is more powerful and consistent across the studies in comparison with that of ischemic postconditioning [12]. This is understandable, as preconditioning is applied before the onset of ischemia and, hence, can utilize the additional innate pathways, both delaying the ischemic injury and attenuating the reperfusion injury. Therefore, either the dose of aspirin of 10 mg/kg used by Li and Kloner [11] might have been insufficient to block preconditioning’s protective mechanisms or the infarct-limiting effect of ischemic postconditioning is fundamentally different from ischemic preconditioning. The present study used an aspirin dose of 30 mg/kg which was half again greater than that used in the Birnbaum et al. postconditioning study [5].

In comparison with local ischemic postconditioning, RIC is more applicable in clinical scenarios, including ACS. Most importantly, RIC can be initiated earlier, before re-opening the culprit artery, thus delaying irreversible damage in ischemic myocardium and, therefore, increasing the volume of salvaged myocardium. It is believed that the protective signal of RIC is transferred from the remote organ to the heart via humoral and neural pathways [13], the neural pathway involving the activation of vagal pre-ganglionic neurons [14]. Therefore, the mechanism of cardioprotection is conceivably not directly dependent of platelet function. Regarding the downstream signaling mechanism of RIC, it differs from that of local ischemic postconditioning. While local postconditioning appears to activate the RISK pathway [10], RIC may recruit the RISK and/or an alternative kinase signaling pathway referred to as the SAFE pathway, depending on the species [15]. Therefore, we hypothesized that if the attenuation of the infarct-limiting effect of ischemic postconditioning is related to platelet function or to hindering RISK pathway activation in the heart by aspirin, then RIC-induced cardioprotection should be preserved in the presence of aspirin.

Indeed, in our study, aspirin did not attenuate the infarct-limiting effect of RIC. Importantly, as the anti-cardioprotective effect of aspirin is dose-dependent, we used the dose of 30 mg/kg, which is higher than the dose used by Birnbaum et al. [5].

Our data might appear to contradict the results obtained by Lieder et al. [7]. In that study, 500 or 1000 mg of aspirin was given to healthy volunteers orally before the RIC procedure. The higher of these doses abrogated the transfer of the infarct-limiting effect to isolated hearts by washed platelets and attenuated the transfer of this effect by plasma dialysate. Conceivably, this discrepancy can be explained by the existence of multiple redundant pathways of RIC [13], supporting the general principle of physiological redundancy. Therefore, in the absence of comorbidities, even if the platelet-specific protection is completely lost by using aspirin, the endogenous cardioprotective phenomenon of RIC can still be preserved, at least partially, via other mechanisms. Specifically, the study by Lieder et al. could not evaluate if aspirin disrupts the neural pathway of the infarct-limiting effect, since that can be observed *in vivo* only. In addition, as discussed by the authors [7], *in* vivo aspirin could impact myocardial responsiveness to cardioprotective signaling, while their study protocol excluded any direct impact of aspirin on the rat myocardium by using repeatedly washed platelets.

The dose of aspirin per unit of weight used by Lieder et al. in humans was lower than that administered to rats in our study. In addition, oral route of administration reduced bioavailability of this drug. A closer comparison of pharmacokinetics and pharmacodynamics of aspirin and its metabolites between rats and humans would be required to match the clinical effects of the doses of aspirin between species, including the effects of RIC. However, this was beyond the scope of our study, which aimed to test the possibility of blocking the infarct-limiting effect of RIC with aspirin in a protocol similar to those used in previous studies in rats [5, 8, 11].

To date, there is no clear opinion on the best protocol of RIC, and whether this optimal protocol can be the same for humans and small animals. Our protocol included four cycles of 5-min left hind limb ischemia, while Lieder et al. were performing three cycles. It cannot be excluded that the effect of a shorter protocol can be more susceptible to any factors abolishing or antagonizing the effects of RIC, even if this shorter protocol provides a significant cardioprotection on its own.

We understand that a relatively short reperfusion period is the limitation of our study, and using larger animal species as well as longer reperfusion periods could provide a more robust conclusion. We believe that a larger, multicenter, more rigorously designed pre-clinical study is warranted to investigate the effect of aspirin on the infarct-limiting and other cardioprotective effects of RIC.

## Supplementary Information

ESM 1 (DOCX 163 kb)
