# Supplementary material for: The Infarct-Limiting Effect of Remote Ischemic Conditioning in Rats Is Not Affected by Aspirin
Source: Cardiovasc Drugs Ther. 2023 Dec 20;39(3):691–5. doi: 10.1007/s10557-023-07541-1 (PMC12116765; doi:10.1007/s10557-023-07541-1)
Supplement: Supplementary file 1 — (DOCX 163 kb) [file 10557_2023_7541_MOESM1_ESM.docx]

**Title: The infarct-limiting effect of remote ischaemic conditioning in rats is not affected by aspirin**

**Authors:** Basalay MV^1^, James M Downey^2^, Davidson SM^1^, Yellon DM^1^

1The Hatter Cardiovascular Institute, University College London, 67 Chenies Mews, London WC1E 6HX, UK

2 Department of Physiology and Cell Biology, College of Medicine, University of South Alabama, Mobile, AL 36688, USA

Corresponding Author: Derek M Yellon

0203 447 9888

[d.yellon@ucl.ac.uk](mailto:d.yellon@ucl.ac.uk)

The Hatter Cardiovascular Institute

67 Chenies Mews

London WC1E 6HX

United Kingdom

Dr Maryna Basalay [m.basalay@ucl.ac.uk](mailto:m.basalay@ucl.ac.uk)

Dr James Downey [jdowney@southalabama.edu](mailto:jdowney@southalabama.edu)

Prof Sean Davidson [s.davidson@ucl.ac.uk](mailto:s.davidson@ucl.ac.uk)

**Article type**: Short communication

**Abstract** (190 words)

**Purpose**: Remote ischaemic conditioning (RIC) has been shown to be a powerful cardioprotective therapy in animal models. However, a protective effect in patients presenting with acute myocardial infarction has failed to be confirmed. A recent preclinical study reported that aspirin which is routinely given to patients undergoing reperfusion therapy blocked the infarct-limiting effect of ischemic postconditioning. The present study was designed to test whether aspirin could also be blocking the infarct-limiting effect of RIC. **Methods:** This was investigated *in vivo* using male Sprague Dawley rats (n = 5 to 6 per group) subjected to either 30-min of regional myocardial ischaemia, followed by 120-min reperfusion, or additionally to a RIC protocol initiated after 20 min myocardial ischaemia. The RIC protocol included four cycles of 5-min hind limb ischaemia interspersed with 5 min reperfusion. Intravenous aspirin (30 mg/kg) or vehicle (saline) was administered after 15 min myocardial ischaemia. **Results:** RIC significantly reduced infarct size (IS) by 47%. Aspirin administration did not affect IS nor did it attenuate the infarct-limiting effect of RIC. **Conclusion:** Aspirin administration in the setting of myocardial infarction is not likely to interfere with the cardioprotective effect of RIC.

**Keywords:** Remote conditioning, aspirin, ischaemia/reperfusion injury.

**Short running title:** Remote conditioning is not affected by aspirin

**Words: 2,151**

**Figures: 1**

**Acknowledgements**

This work was supported by funding from the British Heart Foundation (PG/19/51/34493).

**E-mail addresses of the co-authors:**

Basalay MV:

[m.basalay@ucl.ac.uk](mailto:m.basalay@ucl.ac.uk)

James M Downey:

[jdowney@southalabama.edu](mailto:jdowney@southalabama.edu)

Davidson SM:

[s.davidson@ucl.ac.uk](mailto:s.davidson@ucl.ac.uk)

**Introduction**

Despite the rapid progress in the development of recanalization techniques and rapid improvement in accessibility of these techniques for patients with acute coronary syndromes (ACS) worldwide, the detrimental effects of reperfusion injury remain a major problem. Currently, remote ischaemic conditioning (RIC) is a potential strategy for the alleviation of reperfusion injury which may be applicable to the clinical setting of ACS. The infarct-limiting effect of RIC has been demonstrated by many research groups and in different animal species [1]. The strength of this effect in pre-clinical research is comparable to that of classical ischaemic preconditioning. It is understandable, therefore, that the potential of using RIC in clinical practice has been appealing. While a number of proof-of-concept clinical trials in patients with acute myocardial infarction have demonstrated heterogenous but generally promising results on RIC’s ability to reduce myocardial damage [2], such protection was not confirmed by a recent large-scale, international, multicentre, randomized clinical trial [3]. This failure has raised an intense discussion on possible factors which may impede the cardioprotective effectiveness of RIC in patients [4]. One possible explanation is that the medications included in standard therapeutic protocols could be blocking RIC’s protection [4]. It has recently been reported that aspirin, a mainstay in the primary care in patients with ACS, blocks the cardioprotection from ischemic postconditioning in animal hearts [5]. Platelets are known to be key players in cardioprotection [6]. They have recently been shown to transport a cardioprotective signal from a remote organ to the heart, and aspirin abolished the transfer of this protective signal with platelets to isolated rodent hearts [7].

The aim of the present study was to evaluate whether aspirin would attenuate the infarct-limiting effect of RIC in our animal model. If that proved to be the case, then it could explain why RIC is not as protective in patients as it is in animal models where aspirin is absent.

**Materials and methods**

All the experiments were performed in accordance with the European Commission Directive 2010/63/EU (European Convention for the Protection of Vertebrate Animals used for Experimental and Other Scientific Purposes) and the UK Home Office (Scientific Procedures) Act (1986) with project approval from the University College London Institutional Animal Care and Use Committee. The animals were group-housed and maintained on a 12-h light cycle (lights on 07:00) and had ad libitum access to water and food.

**Animals:** Male Sprague Dawley rats of 190-210g weight, N=23 in total, were purchased from Charles River Laboratories and allowed to acclimatize under standard conditions for at least 1 week. The weight of the rats by the time of their inclusion into the experiment was 250-300 g.

**Ischaemia/reperfusion model:** Rats were anaesthetised with isoflurane: 4% isoflurane for induction and 3-3.75% for maintenance. The maintenance dose was selected on the basis of complete absence of pedal reflex and spontaneous breathing, starting from 2.5% and being increased by 0.25% every 5 min if required. The rats were intubated, their right common carotid artery and left jugular vein cannulated, and the chest was opened by dissecting 2 ribs to the left of the sternum. The heart was then exposed using a chest retractor. After a 10-min stabilisation period, the arterial blood was collected into a capillary tube to measure blood pH. If required, the parameters of ventilation were adjusted, with a re-check of the pH after 10 min. The left anterior descending coronary artery (LAD) was then ligated with a polypropylene suture, needle size 5-0, for 30 min after which it was reperfused for 2 h (Figure 1a). Cling film was used to cover the opening of the chest throughout the experiment to prevent cooling and drying of the heart. Blood pressure and heart rate were recorded throughout the experiment, and body temperature maintained at 36.4 to 37.5°C. At the end of the reperfusion period, the LAD was re-occluded and the hearts perfused, via the jugular vein, with 5% Evan’s blue dye to delineate the area at risk (AAR). The heart was than rapidly excised, the right ventricle removed, and the left ventricle frozen at -80°C and sliced into 6-7 slices of equal thickness. Each slice was scanned from both sides to obtain the images of the AAR. After that, the infarcted myocardium was detected by incubating the frozen heart slices with 1% 2,3,5-triphenyltetrazolium chloride in Tris buffer (pH 7.4) for 15 min at 37°C and fixing in 4% formalin for 24 hours. Finally, the slices were scanned, again from both sides, and each slice was weighed. The AAR and infarct size (IS) were planimetrically evaluated using ImageJ (<https://imagej.nih.gov/ij/>). The weight of AAR was normalised to the weight of the left ventricle. IS was expressed as a proportion of the weight of the infarcted myocardium to the weight of the AAR. The template used for calculations of the IS and the AAR is presented in the Supplement.

**Remote ischaemic conditioning:** RIC was initiated at the 20^th^ min of ischaemia and performed using 4 cycles of 5-min left hind limb ischemia interspersed with 5-min reperfusion, using an inflatable 12-mm cuff (IVM, USA), which was inflated to 200 mmHg and subsequently deflated (Figure 1a). The efficiency of blood cessation with hind limb cuff inflation in rats had previously been confirmed.

**Drug administration:** The dose of aspirin of 30 mg/kg was chosen based on the previous studies, showing that 20 mg/kg of aspirin abolished the infarct-limiting effect of local ischaemic postconditioning [5], and that this effect of aspirin was dose-dependent [8]. In our study, aspirin (Sigma-Aldrich) was dissolved in 1.5 ml of saline at 37°C directly before each of the experiments. This solution was administered as an intravenous bolus over 3 minutes, finishing at the 15^th^ min of ischaemia (Figure 1a). The groups not requiring the administration of aspirin received the same volume of saline warmed to 37°C, over the same time window.

**Randomization and blinding:** Rats were randomly allocated to the experimental groups. The experimenter was not blinded to the experimental groups, however, IS was subsequently evaluated in heart slices by an investigator who was blinded to the treatment.

**Statistical analysis:** The obtained data were analysed with RStudio (<https://cran.r-project.org>, version 2023.06.1). The normality of distribution within the groups was evaluated with Shapiro-Wilk, Kolmogorov-Smirnov, and Pearson tests. The groups were compared using Kruskal-Wallis test, followed by Dunn's Multiple Comparison post-hoc test with Bonferroni correction method. The non-parametric test was chosen based on the presence of outlying IS values in two groups (Figure 1b), as well as due to the small number of animals (5 to 6) per group. Adjusted values of P < 0.05 (P.adj) were considered statistically significant. Data are reported as median, 25^th^ and 75^th^ percentiles (IQR). The format of presenting the data is: median [IQR, 25^th^-75^th^ percentile].

**Results**

None of the animals died prior to completion of the protocol nor were any excluded. There were no differences in haemodynamic parametres between the groups at any time point of the experimental protocol. AAR were also comparable in all the experimental groups. All the raw data are presented in Supplement.

IS in the control group was 43% [IQR, 42-46] % (Figure 1b). RIC, as expected, reduced IS: 23% [IQR, 15-33] % (P.adj < 0.05 vs. Control). Aspirin alone had no effect on IS: 43% [IQR, 39-48] %. Similarly, aspirin had no effect on the infarct-limiting effect of RIC (Aspirin+RIC group): IS = 15% [IQR, 12-19] % (P.adj < 0.05 vs. Aspirin).

**Discussion**

Platelets are known to play the key role in the pathophysiology of ACS, as their activation and aggregation contribute to initiation and propagation of acute ischaemia/reperfusion injury. However, healthy platelets may also activate the cardioprotective RISK pathway (reviewed in [9]), a universal signalling pathway shared by a number of cardioprotective therapies, including ischaemic postconditioning [10]. Interestingly, aspirin has recently been demonstrated to abolish the infarct-limiting effect of local ischaemic postconditioning in a rat model of ischaemia/reperfusion [5]. The exact mechanism of this has not been investigated. We believe that RISK pathway inhibition by aspirin may be one of the plausible explanations. Interestingly, in another study, the infarct-limiting effect of classical ischaemic preconditioning was not affected by aspirin [11]. However, the dose of aspirin in that study was half that used by Birnbaum et al. This may be important, as the attenuation of statin-induced cardioprotection by aspirin was shown to be dose-dependent [8]. In addition, the infarct-limiting effect of classical ischaemic preconditioning is more powerful and consistent across the studies in comparison with that of ischaemic postconditioning [12]. This is understandable, as preconditioning is applied before the onset of ischaemia, and hence, can utilize the additional innate pathways, both delaying the ischaemic injury and attenuating the reperfusion injury. Therefore, either the dose of aspirin of 10 mg/kg used by Li and Kloner [11] might have been insufficient to block preconditioning’s protective mechanisms or the infarct-limiting effect of ischaemic postconditioning is fundamentally different from ischaemic preconditioning. The present study used an aspirin dose of 30mg/kg which was half again greater than that used in the Birnbaum et. al. postconditioning study [5].

In comparison with local ischaemic postconditioning, RIC is more applicable in clinical scenarios, including ACS. Most importantly, RIC can be initiated earlier, before re-opening the culprit artery, thus delaying irreversible damage in ischaemic myocardium, and therefore, increasing the volume of salvaged myocardium. It is believed that the protective signal of RIC is transferred from the remote organ to the heart via humoral and neural pathways [13]; the neural pathway involving the activation of vagal pre-ganglionic neurons [14]. Therefore, the mechanism of cardioprotection is conceivably not directly dependent of platelet function. Regarding the downstream signalling mechanism of RIC, it differs from that of local ischaemic postconditioning. While local postconditioning appears to activate the RISK pathway [10], RIC may recruit the RISK and/or an alternative kinase signaling pathway referred to as the SAFE pathway, depending on the species [15]. Therefore, we hypothesized that if the attenuation of the infarct-limiting effect of ischaemic postconditioning is related to platelet function or to hindering RISK pathway activation in the heart by aspirin, then RIC-induced cardioprotection should be preserved in the presence of aspirin.

Indeed, in our study, aspirin did not attenuate the infarct-limiting effect of RIC. Importantly, as the anti-cardioprotective effect of aspirin is dose-dependent, we used the dose of 30 mg/kg, which is higher than the dose used by Birnbaum et al. [5].

Our data might appear to contradict the results obtained by Lieder et al. [7]. In that study, 500 or 1000 mg of aspirin was given to healthy volunteers orally before the RIC procedure. The higher of these doses abrogated the transfer of the infarct-limiting effect to isolated hearts by washed platelets and attenuated the transfer of this effect by plasma dialysate. Conceivably, this discrepancy can be explained by the existence of multiple redundant pathways of RIC [13], supporting the general principle of physiological redundancy. Therefore, in the absence of comorbidities, even if the platelet-specific protection is completely lost by using aspirin, the endogenous cardioprotective phenomenon of RIC can still be preserved, at least partially, via other mechanisms. Specifically, the study by Lieder et al. could not evaluate if aspirin disrupts the neural pathway of the infarct-limiting effect, since that can be observed *in vivo* only. In addition, as discussed by the authors [7], *in* vivo aspirin could impact myocardial responsiveness to cardioprotective signalling, while their study protocol excluded any direct impact of aspirin on the rat myocardium by using repeatedly washed platelets.

The dose of aspirin per unit of weight used by Lieder et al. in humans was lower than that administered to rats in our study. In addition, oral route of administration reduced bioavailability of this drug. A closer comparison of pharmacokinetics and pharmacodynamics of aspirin and its metabolites between rats and humans would be required to match the clinical effects of the doses of aspirin between species, including the effects of RIC. However, this was beyond the scope of our study, which aimed to test the possibility of blocking the infarct-limiting effect of RIC with aspirin in a protocol similar to those used in previous studies in rats [5, 8, 11].

To date, there is no clear opinion on the best protocol of RIC, and whether this optimal protocol can be the same for humans and small animals. Our protocol included 4 cycles of 5-min left hind limb ischemia, while Lieder et al. were performing 3 cycles. It cannot be excluded, that the effect of a shorter protocol can be more susceptible to any factors abolishing or antagonizing the effects of RIC, even if this shorter protocol provides a significant cardioprotection on its own.

We understand that a relatively short reperfusion period is the limitation of our study, and using larger animal species as well as longer reperfusion periods could provide a more robust conclusion. We believe that a larger, multicentre, more rigorously designed pre-clinical study is warranted to investigate the effect of aspirin on the infarct-limiting and other cardioprotective effects of RIC.

**References:**

1. Bromage DI, Pickard JMJ, Rossello X, et al. Remote ischaemic conditioning reduces infarct size in animal in vivo models of ischaemia-reperfusion injury: a systematic review and meta-analysis. Cardiovasc Res. 2017;113:288–97.

2. Man C, Gong D, Zhou Y, Fan Y. Meta-analysis of remote ischemic conditioning in patients with acute myocardial infarction. Scientific Reports 2017;7:1–9.

3. Francis R, Chong J, Ramlall M, et al. Effect of remote ischaemic conditioning on infarct size and remodelling in ST-segment elevation myocardial infarction patients: the CONDI-2/ERIC-PPCI CMR substudy. Basic Res Cardiol. 2021;116:59.

4. Bell RM, Basalay M, Bøtker HE, et al. Remote ischaemic conditioning: defining critical criteria for success—report from the 11th Hatter Cardiovascular Workshop. Basic Res Cardiol. 2022;117:39.

5. Birnbaum Y, Ye R, Ye Y. Aspirin Blocks the Infarct-Size Limiting Effect of Ischemic Postconditioning in the Rat. Cardiovasc Drugs Ther. 2023;37:221–4.

6. Davidson SM, Andreadou I, Barile L, et al. Circulating blood cells and extracellular vesicles in acute cardioprotection. Cardiovasc Res. 2019;115:1156–66.

7. Raphael Lieder H, Tsoumani M, Andreadou I, Schrör K, Heusch G, Kleinbongard P. Platelet-Mediated Transfer of Cardioprotection by Remote Ischemic Conditioning and Its Abrogation by Aspirin But Not by Ticagrelor. Cardiovasc Drugs Ther. 2022;37:865–76.

8. Birnbaum Y, Lin Y, Ye Y, et al. Aspirin before reperfusion blunts the infarct size limiting effect of atorvastatin. Am J Physiol Heart Circ Physiol. 2007;292:H2891-7.

9. Hałucha K, Rak-Pasikowska A, Bil-Lula I. Protective Role of Platelets in Myocardial Infarction and Ischemia/Reperfusion Injury. Cardiol Res Pract. 2021:5545416.

10. Barsukevich V, Basalay M, Sanchez J, et al. Distinct cardioprotective mechanisms of immediate, early and delayed ischaemic postconditioning. Basic Res Cardiol. 2015;110:452.

11. Li Y, Kloner RA. Cardioprotective effects of ischaemic preconditioning are not mediated by prostanoids. Cardiovasc Res. 1992;26:226–31.

12. Skyschally A, van Caster P, Iliodromitis EK, Schulz R, Kremastinos DT, Heusch G. Ischemic postconditioning: experimental models and protocol algorithms. Basic Res Cardiol. 2009;104:469–83.

13. Pickard JMJ, Davidson SM, Hausenloy DJ, Yellon DM. Co-dependence of the neural and humoral pathways in the mechanism of remote ischemic conditioning. Basic Res Cardiol. 2016;111:50.

14. Mastitskaya S, Marina N, Gourine A, Gilbey MP, Spyer KM, Teschemacher AG, et al. Cardioprotection evoked by remote ischaemic preconditioning is critically dependent on the activity of vagal pre-ganglionic neurones. Cardiovasc Res. 2012;95:487–94.

15. Skyschally A, Gent S, Amanakis G, Schulte C, Kleinbongard P, Heusch G. Across-Species Transfer of Protection by Remote Ischemic Preconditioning With Species-Specific Myocardial Signal Transduction by Reperfusion Injury Salvage Kinase and Survival Activating Factor Enhancement Pathways. Circ Res. 2015;117:279–88.

**Statements and Declarations**

**Funding**: This work was supported by funding from the British Heart Foundation (grant number PG/19/51/34493)

**Competing Interests**: The authors declare that they have no relevant financial or non-financial interests to disclose.

**Author contributions:** MB carried out the experiments described in this report under experimental planning and guidance from SMD and DMY and discussion from JMD. MB drafted the manuscript, and all authors contributed to the final version.

**Ethical Statement**: All applicable international, national, and/or institutional guidelines for the care and use of animals were followed. All procedures performed in studies involving animals were in accordance with the ethical standards of the institution or practice at which the studies were conducted. This article does not contain any studies with human participants performed by any of the authors.

**Figure Legend**

**Figure 1**

**Aspirin does not attenuate the infarct-limiting effect of remote ischaemic conditioning.**

1. The experimental protocol. 30-min myocardial regional ischaemia was followed by 2 hours of reperfusion. Remote ischaemic conditioning (RIC) was initiated on the 20^th^ min of myocardial ischaemia. It was performed as four 5-min cycles of limb ischaemia, interspersed with 5-min intervals of reperfusion. Aspirin was dissolved in saline and administered into the left jugular vein as a 3-min bolus, finishing on the 15^th^ min of myocardial ischaemia. Control and RIC groups received the same volume of saline. The number of animals in the groups was the following: Control (n = 6), Aspirin (n = 5), RIC (n = 6), Aspirin + RIC (n = 6).
2. Infarct sizes (IS) in the experimental groups. The scatterplot represents individual data for each animal, and the boxplot illustrates medians, 25^th^ and 75^th^ percentiles for the groups. The groups were compared using Kruskal-Wallis, followed by Dunn's Multiple Comparison post-hoc test. P values were adjusted using Bonferroni correction method (P.adj). Values of P.adj < 0.05 were considered statistically significant.
